# Supplementary material for: Analysis of the chromatin landscape and RNA polymerase II binding at SIN3-regulated genes
Source: Biol Open. 2023 Nov 10;12(11):bio060026. doi: 10.1242/bio.060026 (PMC10651107; doi:10.1242/bio.060026)
Supplement: Supplementary information [file biolopen-12-060026-s1.pdf]

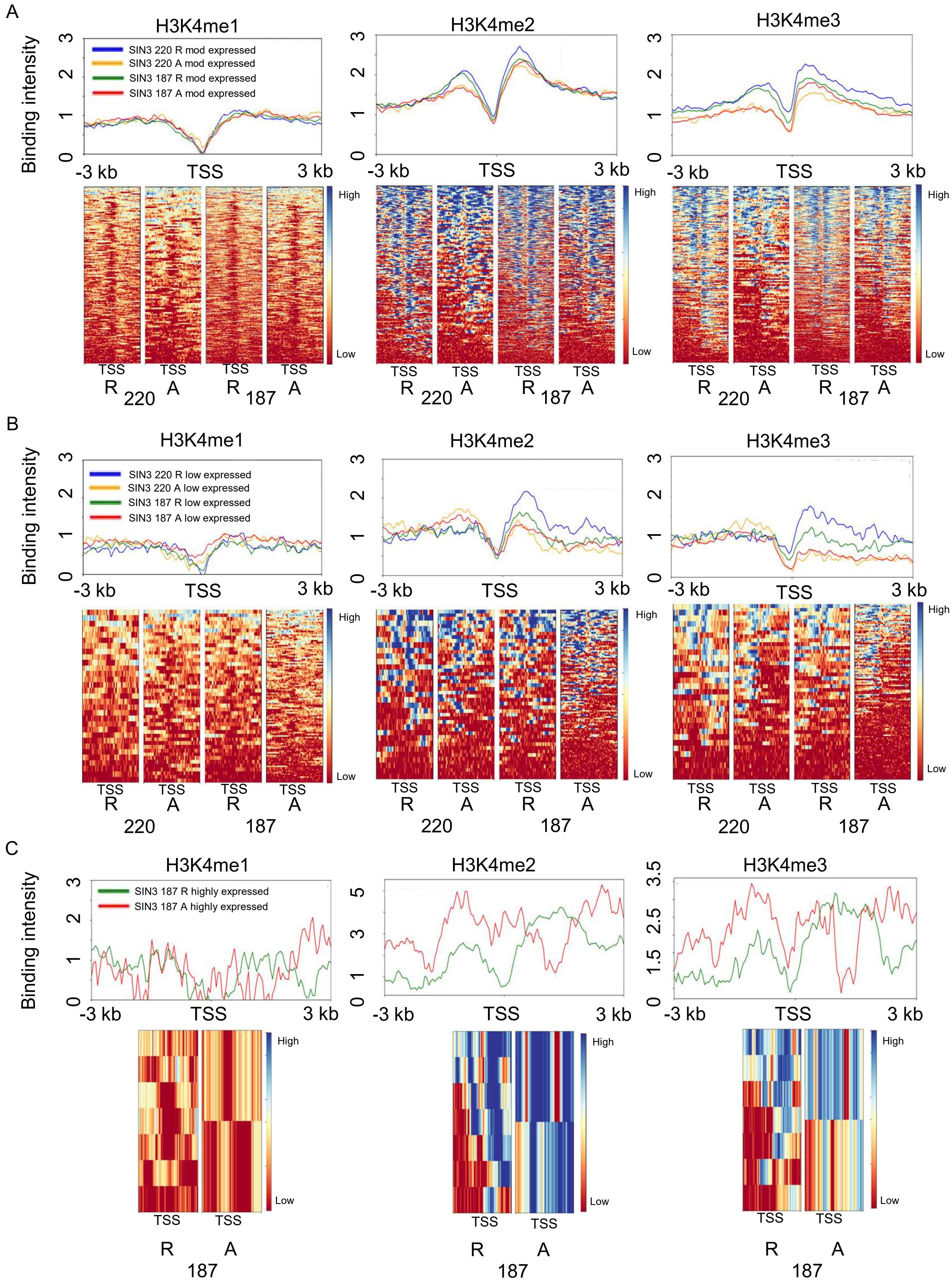

Biology Open • Supplementary information

Fig. S1. H3K4 methylation at genes regulated by the SIN3 isoforms and separated based on expression levels. A. H3K4me1, H3K4me2, and H3K4me3 enrichment was mapped to SIN3 220 and SIN3 187 regulated genes that are moderately expressed. B. H3K4me1, H3K4me2, and H3K4me3 enrichment were mapped to SIN3 220 and SIN3 187 regulated genes that are lowly expressed. C. H3K4me1, H3K4me2, and H3K4me3 enrichment were mapped to SIN3 187 regulated genes that are highly expressed. Heat maps and binding profiles were generated spanning genes from -3 kb to 3 kb. R = repressed, A = activated, TSS = transcription start site.

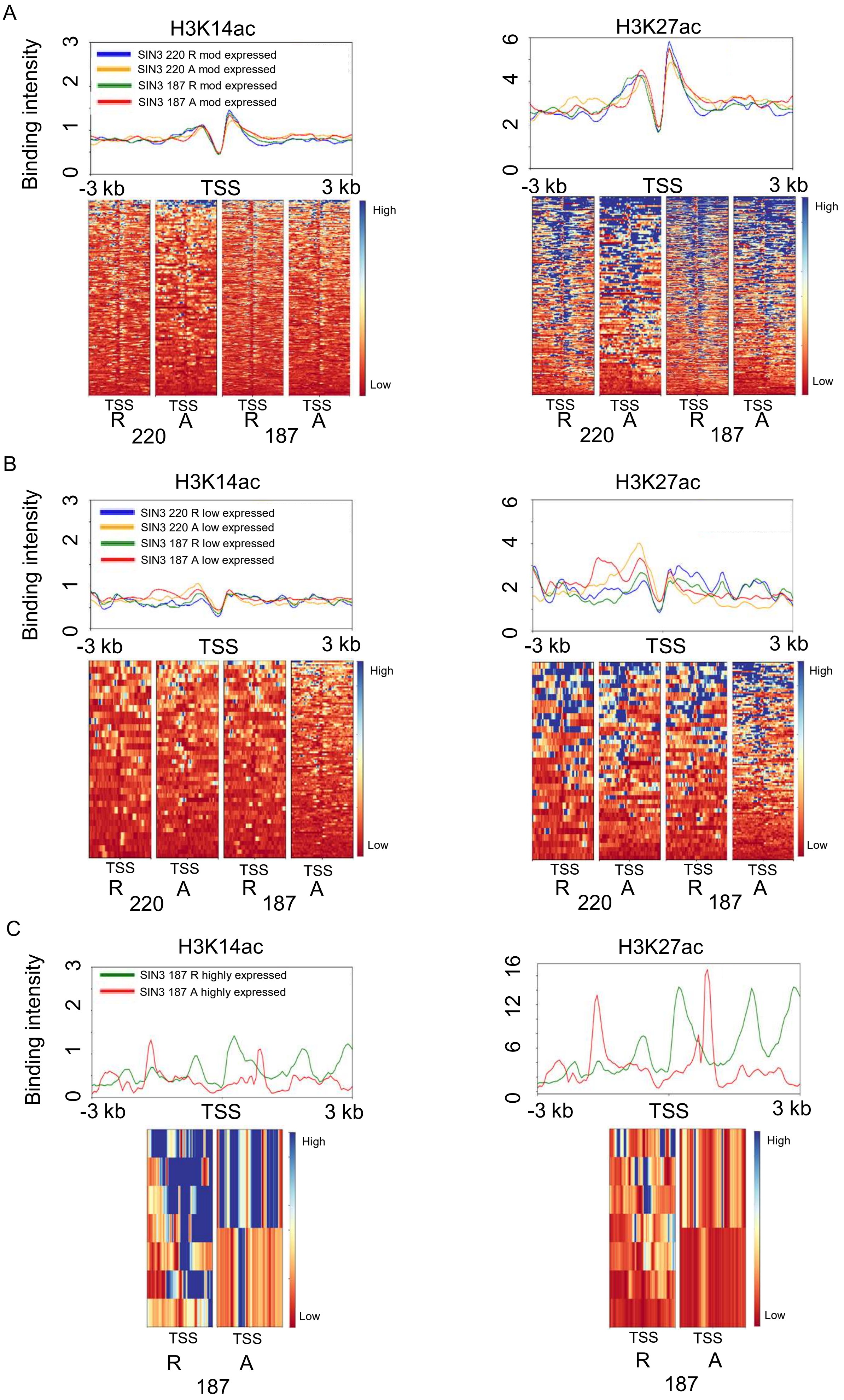

Fig. S2. H3K14 and H3K27 acetylation at genes regulated by the SIN3 isoforms and separated based on expression levels. A. H3K14ac and H3K27ac enrichment were mapped to SIN3 220 and SIN3 187 regulated genes that are moderately expressed. B. H3K14ac and H3K27ac enrichment were mapped to SIN3 220 and SIN3 187 regulated genes that are lowly expressed. C. H3K14ac and H3K27ac enrichment were mapped to SIN3 187 regulated genes that are highly expressed. Heat maps and binding profiles were generated spanning genes from -3 kb to 3 kb. R = repressed, A = activated, TSS = transcription start site.

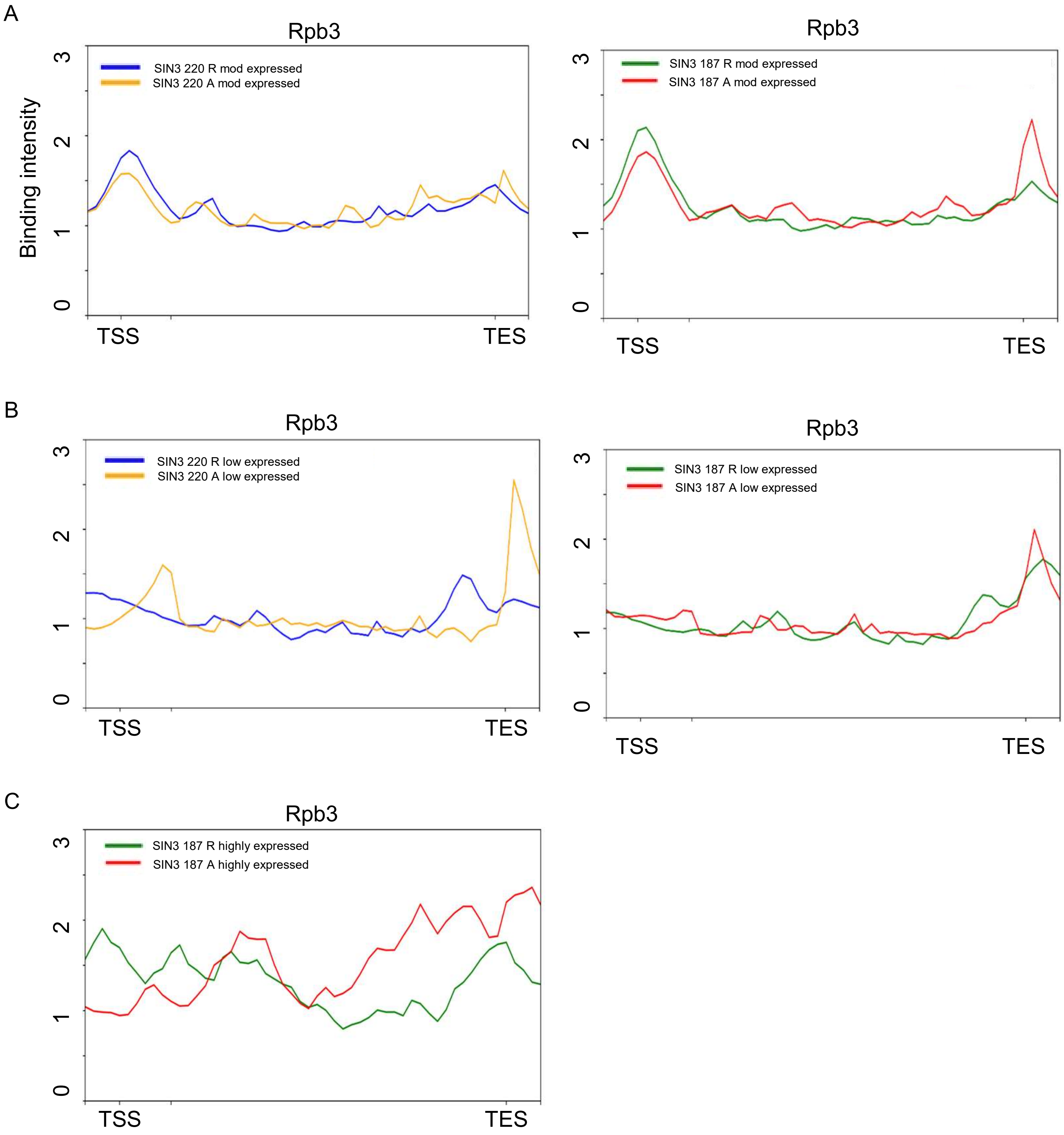

Fig. S3. Rbp3 binding at SIN3-regulated genes separated by expression levels. A. RNA Pol II subunit Rpb3 was mapped against moderately expressed genes that are regulated by both SIN3 isoforms. B. RNA Pol II subunit Rpb3 was mapped against lowly expressed genes that are regulated by both SIN3 isoforms. C. RNA Pol II subunit Rpb3 was mapped against highly expressed genes that are regulated by SIN3 187. R = repressed, A = activated, TSS = transcription start site, TES = transcription end site.

Biology Open • Supplementary information
